# Laryngeal malformations in the Richieri Costa and Pereira syndrome with airway obstruction

**DOI:** 10.1590/S1808-86942011000100025

**Published:** 2015-10-19

**Authors:** Patrícia Barcelos Ogando, Fábio Pires, Rita Carolina Pozzer Krummenauer, Marcus Vinícius Martins Collares, José Faibes Lubianca Neto

**Affiliations:** 1 Physician, medical resident at the Otorhinolaryngology Unit, Health Science Federal University of Porto Alegre (Universidade Federal de Ciências da Saúde de Porto Alegre - UFCSPA). Hospital system of the Porto Alegre Holy House of Mercy (Santa Casa de Porto Alegre - CHSCPA); 2 Physician, medical resident at the Otorhinolaryngology Unit, Professor Edmundo Vasconcelos Hospital, SP; 3 UFCSPA. Otorhinolaryngologist of the Pediatric Otorhinolaryngology Unit, HCSA; 4 Craniomaxillofacial plastic surgeon, doctor in medicine and surgery, Barcelona University (Universidad de Barcelona), professor of the graduate course of medicine, Rio Grande do Sul Federal University (Universidade Federal do Rio Grande do Sul); 5 Rio Grande do Sul Federal University, adjunct professor of otorhinolaryngology, Department of Surgery, UFCSPA. Head of the Pediatric Otorhinolaryngology Unit, HCSA, CHSCPA

**Keywords:** craniofacial dysostosis, laryngomalacia, pierre robin syndrome

## INTRODUCTION

The Richieri-Costa-Pereira syndrome was first described in 1992 in Brazil; it consists of a form of acrofacial dysostosis. It displays mainly low stature, Pierre Robin's sequence (micrognathia, glossoptosis and cleft palate), cleft mandible, and anomalies of the hand and feet such as congenital club foot, clinodactyly of the 5th finger, and underdeveloped thumb. Neuropsychological development is generally within normal limits.^1^

In 1996, Tabith et al.^2^ described the laryngeal findings of this syndrome: short and rounded larynx, absent epiglottis, edema of the aryepiglottic folds, impossibility of visualizing the arytenoids, and presence of a membrane on the posterior wall of the supraglottic larynx. According to previous case reports, these changes account for altered voice quality (soprosity) and other phonation disorders. Airways were unobstructed in all of these cases.

The authors report a case and point to the degree of retrognathia, agenesis of the epiglottis, and involvement of airways that made it impossible to remove the tracheostomy tube.

## CASE REPORT

**Figure 1:**
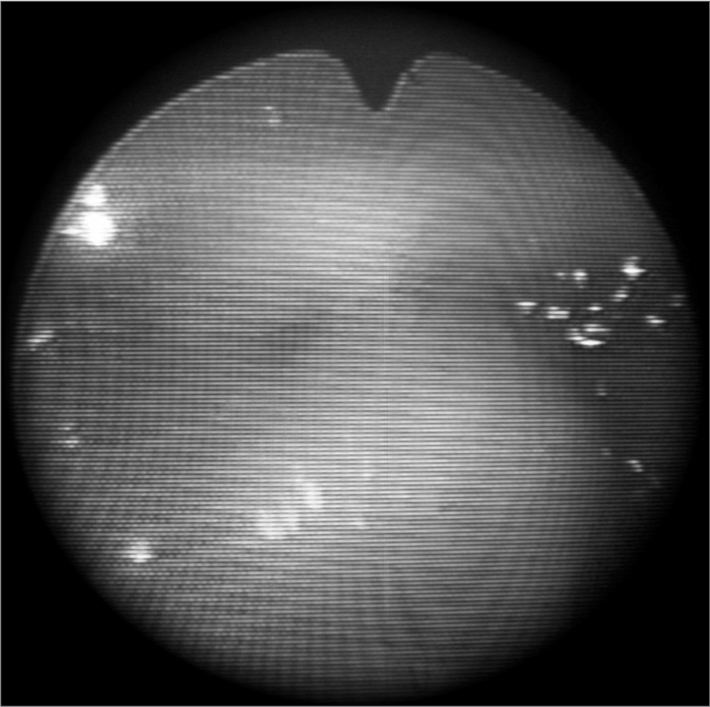
Supraglottic view of the larynx - Absent epiglottis: the arytenoids are seen below and the base of tongue is seen above.

The patient was a child born by cesarean section at 37 weeks gestational age, weighing 2,340 g, measuring 44 cm, with a cephalic perimeter of 32 cm; the 1 and 5 minute Apgar scores were 8 and 9. The mother was primiparous, aged 24 years at the time of birth, whose parents were second degree cousins; there was no family history of genetics diseases. Severe micrognathia, a cleft mandible, and altered clavicle and lower/upper limbs were found on the physical examination. Nasofibrolaryngoscopy revealed a retropositioned tongue that occluded the glottis; the epiglottis was absent and there was severe laryngomalacia that resulted in respiratory failure. A tracheostomy was carried out because of this condition, and the patient remained in hospital until the seventh month of life; during this period the patient had several respiratory events. While still in hospital, mandible distraction surgery followed by cranial bone grafting to anteriorize the mandible and unobstruct the airways for removal of the tracheostomy tube was done. These procedures proved insufficient to maintain the airways pervious, and the patient remained with the tracheostomy tube. Placing a fenestrated tube with a speech valve was also unsuccessful, as the cleaning procedures caused excessive and repeated bleeding, and a suprastomal granuloma due to long term use of the tube resulted in obstruction of the stoma, which made inspiration even more difficult. At age 2 years and 4 months, an external compression of the main left bronchus was observed in a routine bronchoscopy. Computed tomography of the thorax and mediastinum revealed a mediastinal mass that exerted pressure on the main left bronchus; a biopsy resulted in a diagnosis of tuberculosis, which was treated successfully. At present, every two months, the metal tracheostomy tube is changed and bronchoscopy is carried out to assess the airways and, if necessary, to cauterize the suprastomal granuloma. Throughout this period, retrognathism and retropositioning of the tongue have remained unchanged; the tongue rests over the vocal folds (the epiglottis is absent), which maintains the airways obstructed (Fig. 1) and precludes removal of the tube.

## DISCUSSION

At this date, 12 cases of the Richieri-Costa have been reported; one is of a non-Brazilian child. The true prevalence of this disease is unknown.^3,^^4^

The incidence in children of consanguineous parents and in siblings of patients and absence of detectable chromosome abnormalities suggest an autosomal recessive condition. It was first thought that this syndrome was a lethal inherited disease in males - the first few cases that were reported did not survive.^1^ In 1993, the same authors reported two new cases where one boy was aged 1 year and the other was aged 14 years (born in 1979).^5^

In 1996 and 2003, Tabith et al. described laryngeal malformations, as follows: small and oval larynx (glottis), hypoplastic or absent epiglottis, hypertrophic aryepiglottic folds, and presence of a fold on the posterior larynx above the glottis. The physical examination showed medialization of the aryepiglottic folds during phonation, which probably conferred protection to lower airways in these last cases. The phonatory disorders reported in the literature suggest incomplete glottal closure, which results in a hoarse and soprous voice.^2,^^6^

Marked retrognathism and retropositioning of the tongue - as seen in the present report - were not observed in prior cases. This condition precludes removal of the tracheostomy tube and results in comorbidities because of its use. The two surgical procedures (mandible distraction and making a neomandible with cranial bone) did not yield satisfactory results.

## FINAL COMMENTS

The present case underlines the laryngeal malformations seen in these children; upper airways are compromised, requiring an adequate assessment and multidisciplinary follow-up.
